# Towards quantitative metagenomics of wild viruses and other ultra-low concentration DNA samples: a rigorous assessment and optimization of the linker amplification method

**DOI:** 10.1111/j.1462-2920.2012.02791.x

**Published:** 2012-09

**Authors:** Melissa B Duhaime, Li Deng, Bonnie T Poulos, Matthew B Sullivan

**Affiliations:** 1 Department of Ecology and Evolutionary Biology, University of ArizonaTucson, AZ, USA; 2 Helmholtz Zentrum München-German Research Center for Environmental Health, Institute of Groundwater EcologyNeuherberg, Germany

## Abstract

Metagenomics generates and tests hypotheses about dynamics and mechanistic drivers in wild populations, yet commonly suffers from insufficient (< 1 ng) starting genomic material for sequencing. Current solutions for amplifying sufficient DNA for metagenomics analyses include linear amplification for deep sequencing (LADS), which requires more DNA than is normally available, linker-amplified shotgun libraries (LASLs), which is prohibitively low throughput, and whole-genome amplification, which is significantly biased and thus non-quantitative. Here, we adapt the LASL approach to next generation sequencing by offering an alternate polymerase for challenging samples, developing a more efficient sizing step, integrating a ‘reconditioning PCR’ step to increase yield and minimize late-cycle PCR artefacts, and empirically documenting the quantitative capability of the optimized method with both laboratory isolate and wild community viral DNA. Our optimized linker amplification method requires as little as 1 pg of DNA and is the most precise and accurate available, with G + C content amplification biases less than 1.5-fold, even for complex samples as diverse as a wild virus community. While optimized here for 454 sequencing, this linker amplification method can be used to prepare metagenomics libraries for sequencing with next-generation platforms, including Illumina and Ion Torrent, the first of which we tested and present data for here.

## Introduction

Microbial processes drive much of the biogeochemistry that fuels the planet (Falkowski *et al*., 2008), and viruses meddle with these microbial processes at the level of the single cell hosts they infect, resulting in modulation of local- and global-scale biogeochemical processes. This has been best demonstrated in the ocean cyanobacteria and their viruses (cyanophages), whose genomes contain metabolically and environmentally significant genes, including genes for photosynthesis (Mann *et al*., 2003; Lindell *et al*., 2004; Millard *et al*., 2004; Sullivan *et al*., 2006), phosphate stress response (Sullivan *et al*., 2005), nitrogen stress response (Sullivan *et al*., 2010), and nucleotide scavenging (Sullivan *et al*., 2005). In model systems, these core photosynthesis genes are expressed (Clokie *et al*., 2006; Lindell *et al*., 2007) and translated into proteins (Lindell *et al*., 2005) during infection, and are predicted to boost phage fitness (Bragg and Chisholm, 2008; Hellweger, 2009). Further, cyanophages shuffle genes in niche-defining host genomic islands (Coleman *et al*., 2006; Kettler *et al*., 2007; Rodriguez-Valera *et al*., 2009), resulting in viral-driven changes of the host cell surface (Avrani *et al*., 2011).

Yet, in most environments, there are few such model systems available, and ultimately a community-scale context is needed to understand the extent to which model system findings are a reliable proxy for wild populations. Researchers commonly turn to whole community sequencing, metagenomics (Handelsman *et al*., 1998), to probe viral and microbial diversity, protein function and population genomics. Many of these studies are hindered by limited biomass, a consequence of targeted genomics [e.g. stable-isotope probing (Neufeld *et al*., 2007), cell sorting (Woyke *et al*., 2009)], low cell density microbial communities (Biddle *et al*., 2008) or, as in virus studies, small target genome sizes. For example, a typical 20 l ocean virus sample yields on the order of 1 pg to 1 ng DNA, while 454 pyrosequencing and Illumina require 1–5 µg for standard library prep, with slightly less DNA necessary for recent methodological advances, such as linear amplified deep sequencing (LADS), which requires 3–40 ng DNA (Hoeijmakers *et al*., 2011) and Nextera,which requires > 50 ng (Marine *et al*., 2011). To date, viral researchers have relied on linker amplification shotgun libraries (LASLs; Breitbart *et al*., 2002; Vega Thurber, 2009) or whole-genome amplification methods [e.g. multiple displacement amplification (MDA); Angly *et al*., 2006; Dinsdale *et al*., 2008] to generate sufficient material. However, the former suffers from cloning biases and does not scale for next-generation sequencing and the latter suffers from stochastic amplification biases, which render the resulting metagenomes non-quantitative (Abulencia *et al*., 2006; Zhang *et al*., 2006; Arriola *et al*., 2007) and can skew a community's taxonomic profile (Yilmaz *et al*., 2010), rendering cross-sample comparison meaningless.

Two recent developments set the stage for progress, particularly in environmental viral genomics. First, a new precipitation method improves aquatic viral concentration efficiencies from < 25% (typical of tangential flow filtration) to nearly 100% (John *et al*., 2011). Second, the Broad Institute recently modified LASL protocols (Breitbart *et al*., 2002) for 454 pyrosequencing (Henn *et al*., 2010). Briefly, in this linker amplification (LA) modification for next-generation platforms, DNA is sheared, blunt-end repaired and linker-ligated, then gel-sized to a narrow size range before PCR amplification to generate greater quantities of the target DNA. Genome sequencing of viral isolates suggested that these features minimize inherent PCR biases (Henn *et al*., 2010), generally thought to be due to heterogeneous fragment lengths and variable primer site annealing.

Here, we further improve upon the LA method through assessment of *sensitivity –* by answering ‘how low can we go?’ with respect to starting DNA concentrations, *efficiency –* by identifying and optimizing steps where sample loss occurs, *accuracy –* by empirically quantifying sequence biases introduced, and *applicability –* by successful application of the method to multiple next-generation sequencing platforms.

## Results and discussion

### LA method optimizations

**Fig. 1:**
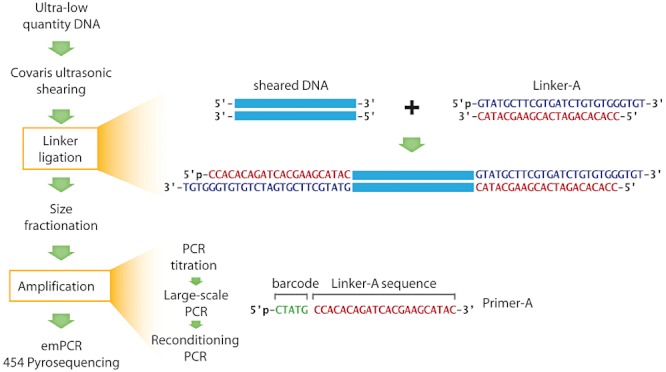
Linker amplification (LA) method schema. This study assesses an optimized LA method, with particular focus on providing new bar-codes in the linker ligation step to facilitate pooling of samples, as well as quantitative evaluation of the impact of amplification on resulting isolate and community DNA genomic sequencing.

Briefly (Fig. 1), extracted DNA is sheared to 400–800 bp using an ultrasonic technique (Covaris). The sheared DNA is end-repaired to facilitate ligation of oligonucleotide linkers. Linker-ligated DNA is then size fractionated (400–800 bp) to target the properly ligated DNA. A small-scale PCR titration is performed to determine the optimal cycle number (lowest number of cycles resulting in a high molecular weight product) for large-scale PCR. A three-cycle reconditioning step is performed to reduce heteroduplexes, increase product yield, and enrich for high molecular weight DNA (Thompson *et al*., 2002) that can be sequenced with next generation sequencers. Here, each step of this LA method was assessed and optimized (Table 1; Table S1), empirically determining the effects of DNA concentration, PCR cycle number, and reconditioning PCR on the resulting datasets generated from each a clonal virus isolate and an environmental viral community (Table 1).

**Table 1:** Summary of treatments studied in linker amplification sequence analysis

Pool	Treatment	Input DNA (ng)	PCR cycles	Barcode (5′–3′)	Linker	Reads (post-QC)
***Pseudoalteromonas* phage H105/1: clonal virus lysate**						
1	cyc15A	10	15	CGACA	CCA CAC AGA TCA CGA AGC ATA C	4 306
	cyc15B			CATAG		1 626
	cyc15C			ATGTA		7 582
	cyc18A	1	18	CGTGT		8 072
	cyc18B			ACGTG		11 889
	cyc18C			TGAGT		12 739
	cyc20A	0.1	20	CTCTA		8 729
	cyc20B			ACTCT		3
	cyc20C			TGCTG		5 186
	cyc25A	0.01	25	CTATG		8 722
	cyc25B			AGCAT		8 006
	cyc25C			TCGCA		6 091
	cyc30A	0.001	30	CTGAG		7 250
	cyc30B			ATCAG		8 201
	cyc30C			TCATA		10 992
2	cyc15rA	10	15 + 3^a^	CGACA	CCA CAC AGA TCA CGA AGC ATA C	1 287
	cyc15rB			CATAG		2 088
	cyc15rC			ATGTA		1 710
	cyc18rA	1	18 + 3	CGTGT		1 183
	cyc18rB			ACGTG		436
	cyc18rC			TGAGT		900
	cyc20rA	0.1	20 + 3	CTCTA		4 431
	cyc20rB			ACTCT		4
	cyc20rC			TGCTG		1 194
	cyc25rA	0.01	25 + 3	CTATG		2 768
	cyc25rB			AGCAT		1 157
	cyc25rC			TCGCA		1 527
	cyc30rA	0.001	30 + 3	CTGAG		1 775
	cyc30rB			ATCAG		5 195
	cyc30rC			TCATA		1 252
	unamp	n/a	No amp	None	ACG AGT GCG TAT ATC GCG AGT CAT	30 279
**Biosphere2 Ocean: environmental virus sample**						
1	B2cyc15A	10	15	CGACA	CCA CAC AGA TCA CGA AGC ATA C	222 421
	B2cyc25A	0.1	25	CAGAT		212 093
	B2cyc15rA	10	15 + 3	ACGTG		119 144
	B2cyc25rA	0.1	25 + 3	TACGA		111 680
2	B2cyc15B	10	15	CGACA	CCA CAC AGA TCA CGA AGC ATA C	261 245
	B2cyc25B	0.1	25	CAGAT		340 488
3	B2cyc15C	10	15	CGACA	CCA CAC AGA TCA CGA AGC ATA C	246 313
	B2cyc25C	0.1	25	CAGAT		310 311
4	unamp A	n/a	No amp	None	ACG AGT GCG TAT ATC GCG AGT CAT	132 639
5	unamp B	n/a	No amp	None	None	160 879

Triplicates are differentiated as A, B and C; reconditioned samples are identified with an ‘r’. When text in a row is blank, refer to the previously listed text; for example, input DNA for each of cyc15A, cyc15B, and cyc15C is 10 ng.

a. Three additional cycles represent the reconditioning PCR.

n/a, not applicable; No amp, no amplification.

First, we designed a new set of 5 bp barcodes to label DNA samples uniquely during amplification and allow pooling of multiple samples on the sequencing plate (Fig. 1, Table 1).

Second, we identified an alternative high-fidelity polymerase (LA TaKaRa HS) to complement that previously used (*Pfu* Turbo HotStart; Henn *et al*., 2010). Both enzymes yield product from starting DNA concentrations as low as 100 fg in only 30–35 PCR cycles (Table S2). However, differences emerged. Based on sensitivity, TaKaRa outperformed *Pfu* for microbial 16S samples and an isolate genome dilution series, while the opposite held true for DNA extracted from a varied collection of ocean virus concentrates (Table S2). However, the sensitivity of *Pfu* came at a cost, as this enzyme amplified a ‘no template control’ at 30 and 35 cycles, while TaKaRa did not (Table S2). Further, in select samples, TaKaRa yielded more product and with a broader size range than *Pfu* (Fig. S1). Finally, while *Pfu* was more sensitive, amplifying some samples that could not be amplified by TaKaRa (Table S2), the LA TaKaRa HS enzyme enriched for sequences that were of extremely low abundance, ‘rares’, in the original sample (addressed below in sequence analysis, as well as in companion paper, Hurwitz *et al*., 2012). In summary, we found that enzyme choice depended on sample type and study question. If one enzyme is unable to amplify a challenging sample, success with the other is likely. Furthermore, the use of LA TaKaRa HS polymerase may be of particular interest to studies targeting components of the rare biosphere (*see Results and discussion: Assessment of systematic biases due to amplification*).

Third, we sought to determine the most efficient and precise post-shearing size selection protocol. Efficiency and precision are necessary as environmental samples often yield ultra-low DNA concentrations, yet next-generation sequencing libraries require significant amounts of DNA, e.g. 1–5 µg, of precise sizes, e.g. 400–800 bp for 454 pyrosequencing (Roche Diagnostics GmbH, 2009a) and 300–450 bp for Illumina (2009; Illumina Sample Preparation Guide).

**Fig. 2:**
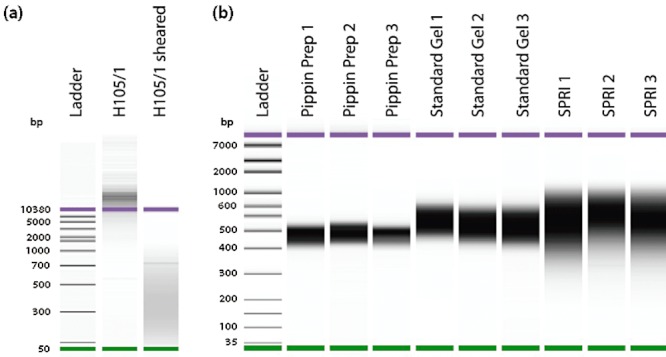
A. Comparison of sheared H105/1 genomic DNA versus unsheared. DNA was run on Agilent chip (DNA 7500 ladder). B. Comparison of size-fractionation methods. Size-fractionation of sheared DNA targeting the 400–600 bp range. DNA was run on Agilent chip (high-sensitivity ladder).

Of the three sizing fractionation methods tested for target recovery efficiency (fraction recovered DNA in target 400–600 bp size range), throughput (ease of applicability to numerous samples simultaneously), and risk of cross-sample contamination, Pippin Prep, an automated optical electrophoretic system that does not require gel extraction, was the most efficient and reproducible (94–96% of input DNA, *n* = 3; Table S3), with the tightest, most specific sizing (Fig. 2B). Pippin Prep and Solid Phase Reversible Immobilization (SPRI), a method based on size-specific capture of DNA by AmPure beads, were equally as high-throughput with low risk of cross-contamination. Yet, SPRI was the least efficient, recovering 46–50% of the targeted size fraction post-shearing (*n* = 3), as it can not be used to bound the upper size range (leaving DNA > 600 bp) due to a suboptimal ratio of PEG:DNA, the mechanism used to remove DNA fragments (Hawkins *et al*., 1994). However, to size fractionate DNA for Illumina libraries (targeting 300–450 bp), the double-SPRI (dSPRI) method has been shown to be a viable method (Rodrigue *et al*., 2010), as it caps both high and low size ranges. Of the three methods tested, standard gel extraction had moderate target recovery efficiency (64–74%). However, this efficiency varies greatly with researcher proficiency and, further, the size selection takes orders of magnitude more time and risks cross-sample contamination. Based on this comparative analysis, we recommend the Pippin Prep automated electrophoretic system to prepare samples for 454 pyrosequencing libraries.

Finally, we quantified the full process post-shearing for seven phage lysates and environmental virus samples and found that the concentration, blunt-end repair, linker ligation and size fractionation (gel-based) was 5.7% ± 2.3% efficient, with respect to total DNA recovery (Table S4; note this calculation is based on gel size fractionation – it is likely that efficiency is higher with the newly assessed Pippin Prep, the most efficient sizing method tested; Table S3). We have found the typical increase in DNA yield due to LA to range from 10- to 1200-fold (Table S5).

### Empirical evaluation of amplification biases

After optimizing the LA method, we sought to determine quantitatively the influence of starting DNA concentration and amplification parameters, such as cycle number and reconditioning (three extra rounds of amplification to reduce heteroduplex formation; Thompson *et al*., 2002), on sequence data from the clonal virus isolate, H105/1, and environmental virus community DNA from the Biosphere2 ocean (B2O; Oracle, AZ).

#### Effect of starting DNA, cycle number and reconditioning on read depth

**Fig. 3:**
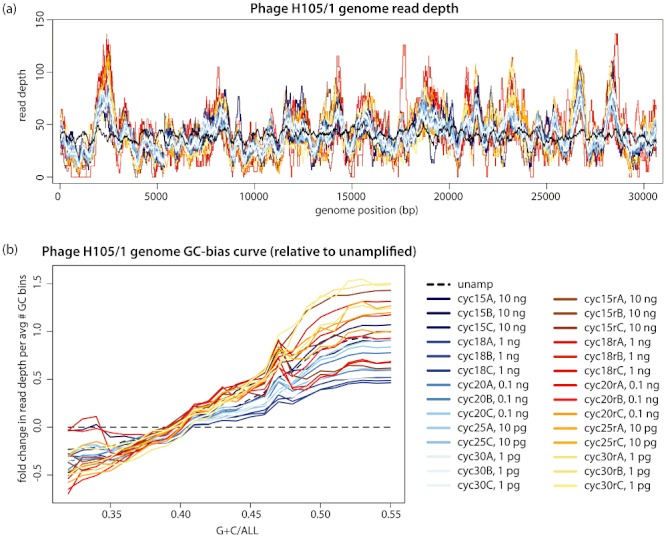
Quantitative evaluation of resulting isolate genome sequencing data. A. Read depth of treatments, as mapped to the Phage H105/1 reference genome: 15–30 PCR cycles, with (red) and without (blue) reconditioning, and unamplified (black) genomic DNA. Counts are scaled by the total number of nucleotides per treatment (Table 1) and multiplied by a factor (1 222 442, the average number of nucleotides in all treatments), to scale to a relatable ‘read depth’ value. B. H105/1 genome ‘GC-bias curve’ representing the relationship between %G + C and read depth, as calculated in a 500 bp sliding window across the genome. Colour scale shared between (A) and (B).

In order to discern which treatments have an effect on read depth, the deviation of amplified read depths from unamplified was compared (all coloured lines of Fig. 3A). There was *no* significant difference between treatments of different starting quantities of DNA and numbers of PCR cycling (*P* = 0.13–0.82, pairwise two-tailed *t*-tests; Table S6). This is in contrast to the popularly used MDA, with which limiting template DNA concentration (1 ng) imposes dramatic representational biases on resulting sequence data (Wu *et al*., 2006). At first pass, there was a significant difference between reconditioned and non-reconditioned treatments (*P* = 1.6E-05). However, this was due to the unintentional reduced sequencing effort of the reconditioned samples (fewer reads causing even some regions of the genome to approach, though never reach, zero coverage; Table 1, Fig. 2A) and subsequent scaling of individual datasets by sequencing effort, which artificially inflates the magnitude of reconditioned read depths. When low coverage areas (< sevenfold) are masked from the genome and the same test performed, there is *no* significant difference between reconditioned and non-reconditioned samples (*P* = 0.33). Aiming for average genome coverage of 15× should help to minimize these low coverage areas and avoid scaling issues that can interfere with cross-dataset comparisons when coverage is disparate. Based on this absence of discernible biases imposed by reconditioning, this step is highly recommended, as it has been shown to minimize heteroduplex formation during amplification (Thompson *et al*., 2002) and will ultimately result in a threefold increase in product yield, an important consideration in low DNA samples.

#### Assessment of systematic biases due to amplification

**Fig. 4:**
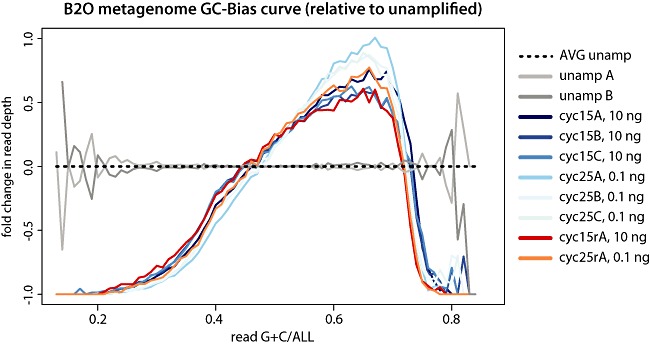
Biosphere2 ocean metagenome ‘GC-bias curve’ representing the %G + C of each read per treatment, relative to the average %G + C of the unamplified treatments.

On the whole, there was notable difference between the amplified and unamplified samples (Fig. 3A), which we hypothesized to result from systematic biases introduced during amplification (e.g. %G + C). Indeed, by normalizing the relative frequency of %G + C-binned reads to those observed in the unamplified treatment, we found the LA method to under-represent regions of the H105/1 genome with < 40% G + C and over-represent regions above, by 0.5- and 1.5-fold respectively (Fig. 3B). Extending this assessment to the B2O metagenome, we found a similar onefold over- and under-representation of reads, relative to that seen in unamplified treatments (Fig. 4). Notably, the %G + C of the B2O metagenome (12–84%) spans a much wider range than H105/1 (31–55%), and is a range characteristic of most sequenced dsDNA virus metagenomes (Fig. S2). At the %G + C extremes seen in the B2O community assemblage, both high and low %G + C reads are under-represented in amplified treatments, a common phenomenon inherent to PCR amplification (Hoeijmakers *et al*., 2011). Regardless, these systematic differences resulting in 0.5- to 1.5-fold biases are a marked improvement over the stochastic representation biases of whole genome amplification, which can lead to 100s-fold (Woyke *et al*., 2009) to 10 000s-fold changes (Zhang *et al*., 2006) and have been shown to render MDA-generated metagenomes non-quantitative (Yilmaz *et al*., 2010). Our optimized LA results are comparable to observations of the new LADS (Hoeijmakers *et al*., 2011) and amplification-free (Kozarewa *et al*., 2009) methods, though these methods require significantly more DNA, 3–40 ng and 100s of ng of input DNA, respectively, which is often unattainable from environmental samples.

Our experimental design allowed us to evaluate variability in replicate metagenome preparations to assess how PCR cycling and reconditioning impacted resultant datasets. We anticipated that increased cycle number might exacerbate the %G + C biases. However, the lack of such a trend indicates that replicate LA datasets prepared with varying amounts of starting DNA (1 pg to 10 ng) and using different cycling conditions are quantitatively comparable (Figs 3 and 4), with few significant differences between treatments (Table S7a). As with the read depth comparison, there was a higher incidence of significant differences between non-reconditioned and reconditioned samples, especially at higher cycle numbers (Table S7b). Indeed, a slight positive trend existed between reconditioned samples and cycle number, which did not exist for the non-reconditioned samples (Fig. S3). Thus, the additional 3-cycle reconditioning step resulted in a 10-fold increase in DNA – and DNA of higher molecular weight (Fig. S1) – at the cost of only slight %G + C bias at higher cycle numbers (Fig. 3B). Importantly, all biases imposed across all treatments are still never more than 0.5- to 1.5-fold (Figs 3 and 4).

**Fig. 5:**
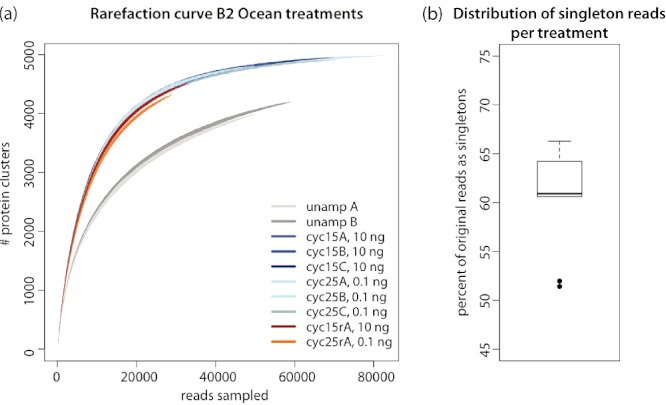
Protein cluster diversity in the Biosphere 2 ocean viral community. A. Rarefaction curve representing the relative sequence diversity of each treatment, as measured by protein clusters (with > 20 sequence members) derived from all amplified and unamplified treatments. Higher diversity in the amplified treatments is likely due to a preferential amplification of the rare biosphere by the LA TaKaRa-HS DNA polymerase used in PCR. B. Boxplot representing the range of percent original reads as singletons in each treatment. The two outliers at 51% are the unamplified treatments.

As a common goal of ecology is cross-community comparisons of diversity, we also examined how LA impacted diversity profiles, represented as rarefaction curves of protein clusters with at least 20 members (Fig. 5A). Regardless of cycle number or reconditioning, the rarefaction curves of amplified treatments were nearly identical, while those from the unamplified treatments were less diverse (Fig. 5). Quantification of ‘singleton’ reads (defined at 90% identity), indicate that rare reads from the original community DNA are enriched for in the amplified treatments (over 62% percent of the original reads; Fig. 5B), with more than 10% fewer singletons in the unamplified treatments.

We hypothesize that this enrichment of rare reads is a feature of the LA TaKaRa polymerase enzyme used during PCR. A related study has shown the enrichment of ‘rares’ does *not* occur in similarly prepared samples where the *Pfu* Turbo master mix was used, with the resultant amplified datasets thus appearing more diverse (data published in companion paper; Hurwitz *et al*., 2012). This trend is supported by Wu and colleagues, who found pyrotag datasets prepared with a *Pfu* polymerase to be less diverse than the same sample amplified with a TaKaRa polymerase, which they attribute, quite generally, to possible differences in the enzymes' processivity or proof-reading (Wu *et al*., 2010). These observations may be related to the fact that during PCR, hydrolytic deamination of dCTP to dUTP in already amplified products can inhibit further amplification of these fragments by *Pfu*, as it is an archaeal family B polymerase known to stall at dU-containing DNA (Lasken *et al*., 1996). However, the *Pfu* Turbo master mix contains a dUTPase enhancement factor (Hogrefe *et al*., 2002) that effectively deaminates dUTP back to dCTP, allowing continued amplification of already amplified fragments and thus presumably maintaining the original relative abundances of DNA fragments in the sample. Thus, one alternate explanation may be that if the TaKaRa enzyme is at all hindered by the presence of dUTP in already amplified templates, the enzyme will preferentially amplify the low abundance, yet to be amplified rare reads, as we observe. These rare reads amplified to detection by TaKaRa do not appear to be artefacts of amplification, as they have sequence similarity to established protein clusters at the same frequency as the abundant reads in the same dataset (Hurwitz *et al*., 2012). Whether this enrichment of rare reads is beneficial or detrimental depends heavily on the downstream application of the sequenced dataset.

#### Application to other sequencing platforms

While the protocol and analysis presented here are developed for 454 pyrosequencing, with minor adjustments this LA protocol is appropriate for samples intended for other sequencing platforms. For instance, shearing conditions can be altered to size templates for shorter read-length sequencing technologies (e.g. 200 bp for Ion Torrent's current 316 chip). Further, template mixtures can be used to sequence with Illumina technologies to circumvent issues with the non-random linker sequence on linker-amplified templates, which causes erroneous base-calling by the Illumina software. To this end, we recently pooled linker amplified DNA from 20 freshwater cyanophage isolates at a ratio of 1:1 with known phi29 DNA template (our template mixture) and successfully generated high quality, full-length Illumina sequence data (Fig. S4; L. Deng and M.B. Sullivan, unpubl. data). Alternate approaches to prep libraries for Illumina sequencing include (i) adding a 3′-U during PCR amplification to the LA linker-containing DNA, such that the linker can be cleaved at the U with, e.g. the USER Enzyme (New England Biolabs; Beverly, MA, USA), followed by an S1 nuclease clean-up of remnant ssDNA, resulting in linker-free DNA for Illumina library prep (described by Rodrigue *et al*., 2009); or (ii) performing the LA method with Illumina linkers. The benefit of the former being that the amplification behaviour of linkers in this study has been rigorously tested and assessed here.

With ever-declining sequencing costs and sequence analysis tools becoming more readily available, metagenomics has become a standard tool for investigating wild communities. As such, it is essential to optimize and popularize robust methods to preserve a quantitative metagenomic signal. The optimized LA method presented here enables a several orders of magnitude increase in DNA yield that results in minimally biased (0.5- to 1.5-fold as compared with unamplified) metagenomes appropriate for comparative analyses from samples with limiting amounts (< 1 ng) of DNA.

## Experimental procedures

The development and optimization of the LA method described below and (detailed in Table S1) are derived from an accumulation of knowledge gained from preparing over 50 varied samples (Tables S2–5; Figs S1–5). The analyses of sequence biases introduced by the method are based on a single phage isolate, *Pseudoaltermonas* phage H105/1 (herein H105/1; Duhaime *et al*., 2011), and an environmental sample from the Biosphere2 ocean (herein B2O; Oracle, AZ, USA).

### Linker amplification

#### Isolation of DNA

To isolate genomic DNA, H105/1 was grown on *Pseudoalteromonas* sp. H105, the lysate was polyethylene glycol/NaCl precipitated and cesium chloride (CsCl) purified, as previously described (Sambrook and Russell, 2001; Duhaime *et al*., 2011). Genomic DNA was extracted using Wizard Prep Resin (Promega; Madison, WI, USA) and mini-columns (Promega) as described by Henn and colleagues (2010). For environmental virus community DNA, 1080 l seawater was 0.2 µm filtered, and the virus-containing filtrate iron chloride precipitated and concentrated per John and colleagues (2011). The resuspended viral concentrate was treated with DNase I (100 units ml^−1^) for 2 h at room temperature, then DNase activity inactivated with 100 mM EDTA/EGTA. This virus preparation was purified on a CsCl gradient (recovering 7.1 × 1011 viruses ml-1 from the ρ = 1.4–1.52 CsCl fraction) (Sambrook and Russell, 2001) and DNA was extracted from 0.6 ml using Wizard Prep Resin (as above). Lysate and environmental DNA was ligated to Adaptor-A (Fig. 1), diluted, and amplified at various PCR cycle numbers (Table 1) per the LA protocol described below.

#### Preparation of sheared, Linker-A Ligated DNA

Covaris Adaptive Focused Acoustics (AFA) was used to shear DNA to 400–800 bp, with the following parameters: 130 µl of DNA in Tris EDTA (TE) buffer (up to 5 µg total DNA), duty cycle of 5%, intensity of 3200 cycles per burst, at 6–8°C for 62 or 120 s, for the Covaris E210 and S2 models respectively. DNA fragment sizes were determined before and after shearing on a DNA 7500 or High Sensitivity chip in the Agilent Bioanalyzer 2100 (Agilent Technologies; Santa Clara, CA, USA). Following shearing, low yield (< 500 ng) samples were concentrated two- to threefold (final volume 35–50 µl) with Amicon Ultra-0.5100 kDa filter units (Millipore; Billerica, MA, USA), according to manufacturer's directions.

Hemi-phosphorylated Linker-A (Fig. 1) was prepared by annealing a synthesized forward linker (5′-phosphorylated-GTA TGC TTC GTG ATC TGT GTG GGT GT-3′; 1.14 mM in TE) to the reverse linker (5′-CCA CAC AGA TCA CGA AGC ATA C-3′; 1.14 mM in TE; *not* phosphorylated in order to promote unidirectional ligation) in a 50 mM NaCl buffer. Equal volumes of forward and reverse linkers were heated to 100°C, slowly cooled on the bench to room temperature, and placed on ice for 5 min. Resultant Linker-A was diluted to 10 µM in TE and stored at −20°C for subsequent use.

Blunt-end repair of sheared DNA, ligation to Linker-A and required cleanup reactions (Fig. 1) were performed as described (Henn *et al*., 2010) using the End-It DNA End-Repair kit (Epicentre Biotechnologies; Madison, WI, USA), Fast-Link DNA Ligation kit (Epicentre Biotechnologies) and Min-Elute Reaction Clean-up kit (Qiagen; Valencia, CA, USA) respectively. Sheared, linker-ligated DNA was then size-fractionated.

H105/1 genomic DNA, 10 µg in 130 µl (77.4 ng µl^−1^) was sheared to 400–600 bp (Covaris) to test three sizing methods in triplicate: gel electrophoresis, SPRI and a digital optical electrophoretic system, Pippin Prep (Sage Science; Beverly, MA, USA). Each method was tested with 13.7 µl of sheared DNA at a concentration of 14.08 ng µl^−1^ (183 ng total DNA). For gel extraction, sheared DNA was run on a 1.5% agarose gel stained with ethidium bromide for 90 min at 80 V. Gel fragments in the 400–600 bp size range were excised, purified to remove agarose (Qiagen MinElute Gell Extraction kit), and eluted in 20 µl EB buffer, as described (Henn *et al*., 2010). SPRI with Agencourt AMPure XP beads (Beckman Coulter; Danvers, MA, USA) was used to remove the less than 400 bp fragments (detailed in *Results and discussion: LA method optimizations*). To specifically retain the desired greater than 400 bp fragments a bead to DNA ratio of 55:100 was used, as determined per Roche (Roche Diagnostics GmbH, 2009a), such that the final reaction contained 13 µl sheared DNA, 7 µl TE buffer, and 11 µl Ampure beads. DNA was eluted in 20 µl EB buffer. Pippin Prep samples were run in pre-cast 2% agarose gel cassettes, pre-stained with ethidium bromide (Sage Science), set to recover the 400–600 bp fragments. DNA was recovered in 39–42 µl final volumes (Table S3). Following each method, DNA accurately retained in the target 400–600 bp range was quantified to determine target recovery efficiency using an Agilent Bioanalyzer 2100 (Agilent Technologies; Santa Clara, CA, USA).

#### Polymerase evaluation, barcode design

Two high fidelity polymerases, *Pfu* Turbo Hotstart (Stratagene; La Jolla, CA, USA) and TaKaRa LA Taq Hotstart (Takara Bio; Shiga, Japan), both with 3′ to 5′ exonuclease (‘proof-reading’) activity and an antibody quencher for hotstart capability, were assessed for efficiency and sensitivity. Both enzyme reactions used 1–2 µl Linker-A ligated and size-fractionated DNA with 0.5 µl (5 pmol) of the 10 µM PCR phos-A primer. For the *Pfu* Turbo Hotstart system, 12.5 µl *Pfu* Turbo Hotstart 2× Master Mix (0.1 U *Pfu* Turbo µl^−1^) was used, while the TaKaRa LA HS system required 2.5 µl 10× PCR buffer, 4.0 µl of 2.5 mM dNTP mix (10 nmol each), and 0.25 µl TaKaRa LS Taq HS (5 U LA TaKaRa µl^−1^). Both reactions were brought to 25 µl with nuclease-free water. For all reactions prepared for the sequence analysis (H105/1 phage genome and B2O environmental DNA), the TaKaRa LA Taq Hotstart system was used.

A series of 5 bp barcodes was added to the ‘phos-A PCR primer’ (5′-p-CCACACAGATCACGAAGCATAC-3′) (Henn *et al*., 2010), such that a sample-specific, unique barcode would be added at the 5′ end of each DNA fragment during amplification (Fig. 1, barcodes listed in Table 1). The barcodes were designed such that (i) no consecutive duplicate nucleotides exist, (ii) barcodes differ by at least 2 nucleotides, (iii) no C appears at the 3′ end, as Linker-A has a 5′ C (Fig. 1), and (iv) no G appears at the 5′ end, as the 454 emPCR primers have a 3′ G (forward: 5′-CGTATCGCCTCCCTCGCGCCATCAG-3′; reverse: 5′-CTATGCGCCTTGCCAGCCCGCTCAG-3′) (Roche Diagnostics GmbH, 2009b).

#### Small-scale PCR titration

To determine the minimum number of PCR cycles needed for amplification of each sample, a small-scale PCR titration was performed with varying cycle numbers [95°C for 2 min, (95°C for 30 s, 60°C for 60 s, 72°C for 90 s) × 15, 18, 20, 25, or 30, 72°C for 10 min]. Generally, three cycle numbers were tested according to the amount of DNA available after ligation and sizing, as a log-linear relationship exists between input DNA and cycle number needed for amplification (Fig. S5). Generally, 1–10 ng DNA samples were run for 15–20 cycles, 0.1–1 ng for 18–25 cycles, 10–100 pg for 22–30 cycles, and less than 10 pg for 25–35 cycles. If DNA was not amplified by 35 cycles, more DNA was added to the PCR reaction (up to 10% of the PCR reaction volume) or the sample was concentrated using AMPure XP beads (80 µl beads to 100 µl DNA) and PCR titration attempted once more.

PCR products were analysed on 1.5% agarose gels with 0.5 ng µl^−1^ ethidium bromide, run in 1× Tris-acetate-EDTA (TAE) buffer at 90 V for 30 min, using 5 µl PCR product mixed with 1 µl 6× Blue/Orange Loading Dye (Promega). Quick Load 100 bp DNA Ladder (New England Biolabs; Beverly, MA, USA), 250 bp DNA Ladder (Invitrogen; Carlsbad, CA, USA), or 1 kB Plus DNA Ladder (Invitrogen) was used for fragment size determination.

#### Large-scale amplification and ‘reconditioning PCR’

Sample DNA was amplified (per PCR protocol above) using the number of cycles determined by the small-scale titration. Depending on quantity of starting DNA, six to ten reactions were performed in this step, resulting in up to 250 µl PCR product, to ensure sufficient DNA for sequencing (1–5 µg per standard 454 pyrosequencing or Illumina library).

To both increase yield and minimize heteroduplex formation, a ‘reconditioning PCR’ step was added, *sensu*Thompson and colleagues (2002). To recondition, the amplified DNA is diluted 10-fold in a fresh PCR reaction mix (200 µl reactions with 2.5 µl TaKaRa LA HS and 20 µl of small-scale PCR product as template, all other reagents in same proportions as in small-scale titration) and amplified for three cycles [95°C for 2 min, (95°C for 30 s, 60°C for 60 s, 72°C for 90 s) × 3, 72°C for 10 min], effectively increasing the primer-to-template ratio by replenishing the reaction with new primer. All PCR products previously generated were reconditioned, resulting in up to 2.5 ml of final reconditioned product, which was then concentrated to 250 µl using Amicon Ultra-0.5100 kDa centrifugal columns, purified with the MinElute PCR Purification (Qiagen) kit, and DNA eluted off the mini-columns with 25–40 µl TE buffer warmed to 80°C. Note that in order to determine the effect of this treatment on resultant sequence data, parallel treatments of phage H105/1 genome and B2O environmental DNA were *not* reconditioned (Table 1). Finally, amplified samples were quantified (Quant-iT Pico Green for dsDNA; Invitrogen) and, where sample pooling was necessary, samples with unique barcodes were mixed in equimolar amounts for sequencing library preparation. Libraries were sequenced using GS FLX Titanium 454 pyrosequencing (no paired-ends) or the Illumina HiSeq (paired-end reads, one channel with raw output of 19.9 GB).

### Sequence analysis

#### Phage genome analysis

Genomic reads were mapped per treatment to the complete H105/1 genome (RefSeq NC_015293) using gsMapper (v 2.5.3). Read depths were divided by the total number of nucleotides per treatment to normalize for sequencing effort. %G + C and average read depths were calculated over a 500 bp sliding window to generate ‘GC bias’ plots.

#### Environmental virus metagenome

Biosphere2 ocean metagenome reads were subjected to quality control to remove reads that (i) contained an ambiguous base, (ii) were at least two standard deviations from the mean sequence length per plate, (iii) were at least two standard deviations from the mean quality score per plate, or (iv) were identified by cd-hit454 (default parameters) as emulsion-PCR replicates. %G + C was calculated per read. For the rarefaction analysis, reads from all treatments were assembled using Velvet (Zerbino and Birney, 2008) with automatic coverage estimation and a *k*-mer length of 21. Genes were predicted on all raw reads and contigs greater than 100 bp using Prodigal (metagenomic gene finding, remainder of parameters as default) (Hyatt *et al*., 2010). Protein clusters with 60% within-cluster identity were built using cd-hit (Li and Godzik, 2006) with word length of 4. Original reads were non-redundantly mapped to protein cluster representative sequences using blastx (Altschul *et al*., 1990) with 60% identity cut-off. Rarefaction curves were generated by sampling protein clusters of > 20 members without replacement, increasing sampling effort in 5000-read steps (100 replicates) using R scripts. To compare the number of ‘rares’, reads were clustered per treatment using cdhit-est (Li and Godzik, 2006) at 90% within-cluster identity, considering both strands, and with a word size of 8. The number of singletons per treatment was defined as the number of reads in single-member clusters.

#### Statistical tests

Tests comparing the relative read depths and %G + C bias between unamplified and amplified treatments were performed using a series of paired two-tailed Student's *t*-tests. To quantify the magnitude of read depth and %G + C bias, the area between the unamplified and amplified curves was calculated by trapezoid integration. Pairwise tests were performed to compare (i) all reconditioned versus all non-reconditioned samples (*n* = 14 pairs), (ii) the reconditioned and non-reconditioned per cycle number (*n* = 3 pairs), (iii) all treatments of a cycle number against each of the remaining cycle number treatments, reconditioned and non-reconditioned combined (*n* = 6 pairs), and (iv) ‘c’ repeated, testing reconditioned and non-reconditioned separately (*n* = 3 pairs).

The LA protocol is available at http://eebweb.arizona.edu/faculty/mbsulli/protocols.htm. All sequence data is available on the CAMERA web portal, tracking number CAM_P_0000912.

## Supporting information

**Fig. S1.** Comparison of DNA polymerase efficiency and effect of reconditioning PCR on two phage lysates, TUSD #20 and #23. LA-TaKaRa yielded more product and with a broader size range than PFU Turbo HotStart. Original PCR product is diluted 10× for input in reconditioning reaction, and thus results in a 10-fold increase in product. Also, note the enrichment for high molecular weight product following reconditioning.

**Fig. S2.** %G + C range of virus communities, as seen using various amplification (linker amplification, phi29 multiple displacement amplification, and linker amplified shotgun libraries) and sequencing platforms (454 pyrosequencing, Sanger sequencing). (A) in-house sequence data, (B) and (C) are identifiable by name and available on the CAMERA web portal, including all metadata and publication references.

**Fig. S3.** The magnitude of difference in %G + C bias between unamplified and amplified treatments was assessed by the magnitude of difference between the integrated area under their %G + C-bias plot curves (Fig. 3). As cycle number increased (and quantity of starting material decreased), there was a slight trend in the deviation of amplified from unamplified treatments.

**Fig. S4.** Quality score profile of Illumina reads from 20 pooled fresh water cyanophage genomes (L. Deng and M.B. Sullivan, unpubl. data) generated from a linker amplified library. Boxplot generated by Fastx Toolbox.

**Fig. S5.** Log-linear relationship between PCR cycle numbers and starting DNA, as it is diluted to extinction. Test was performed with dilution of a single phage genome, H105/1.

**Table S1.** Detailed break-down of linker amplification method, including time and cost estimates from sample to sequence for a typical 20 l aquatic virus sample.

**Table S2.** Comparison of DNA polymerase sensitivity and specificity.

**Table S3.** Comparison of sheared DNA size-fractionation techniques.

**Table S4.** Evaluation of linker amplification efficiency.

**Table S5.** Increase in DNA as a result of linker amplification, from both environmental and phage lysate DNA preps.

**Table S6.** Two-tailed paired Student&apos;s *t*-tests comparing the integrated areas between unamplified read depth curve and curve of each amplified treatment.

**Table S7.** Two-tailed paired Student&apos;s *t*-tests comparing the integrated areas between unamplified %G + C bias curve of unamplified and each amplified treatment for H105/1.
