# Supplementary material for: Towards quantitative metagenomics of wild viruses and other ultra-low concentration DNA samples: a rigorous assessment and optimization of the linker amplification method
Source: Environ Microbiol. 2012 Sep;14(9):2526–37. doi: 10.1111/j.1462-2920.2012.02791.x (PMC3466414; doi:10.1111/j.1462-2920.2012.02791.x)

Supplementary Figure 1. Comparison of DNA polymerase efficiency and effect of reconditioning PCR on two phage lysates, TUSD #20 and #23. LA-TaKaRa yielded more product and with a broader size range than PFU Turbo HotStart. Original PCR product is diluted 10X for input in reconditioning reaction, and thus results in a 10-fold increase in product. Also, note the enrichment for high molecular weight product following reconditioning.

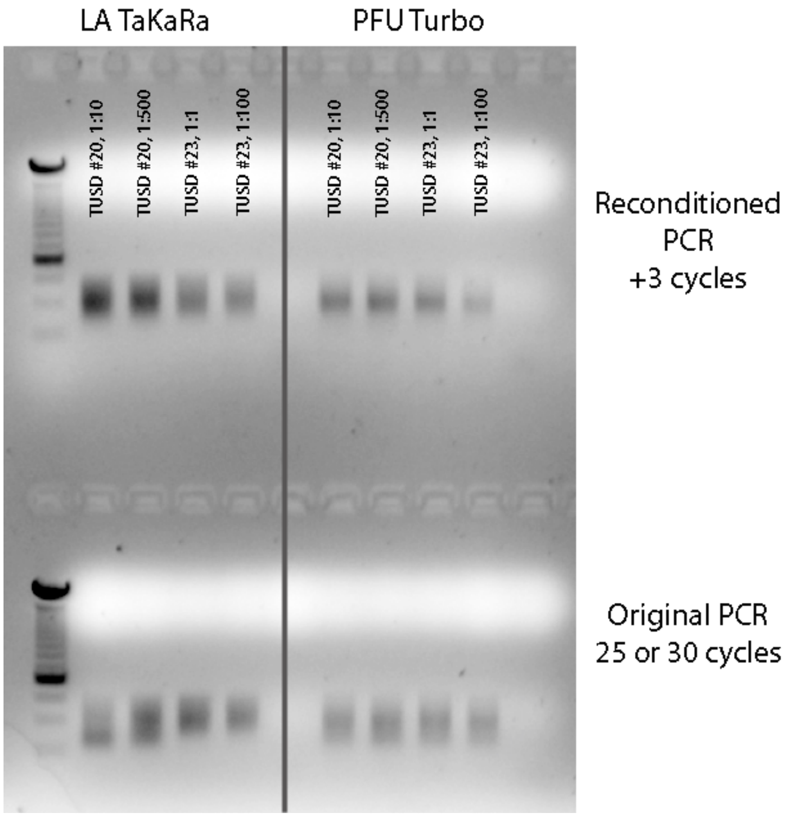

Supplement: Supplementary file 1 [file emi0014-2526-SD1.pdf]
