# Supplementary material for: Towards quantitative metagenomics of wild viruses and other ultra-low concentration DNA samples: a rigorous assessment and optimization of the linker amplification method
Source: Environ Microbiol. 2012 Sep;14(9):2526–37. doi: 10.1111/j.1462-2920.2012.02791.x (PMC3466414; doi:10.1111/j.1462-2920.2012.02791.x)

Supplementary Figure 2. %G+C range of virus communities, as seen using various amplification (linker amplification, phi29 multiple displacement amplification, and linker amplified shotgun libraries) and sequencing platforms (454 pyrosequencing, Sanger sequencing). (a) in-house sequence data, (b) and (c) are identifiable by name and available on the CAMERA web portal, including all metadata and publication references.

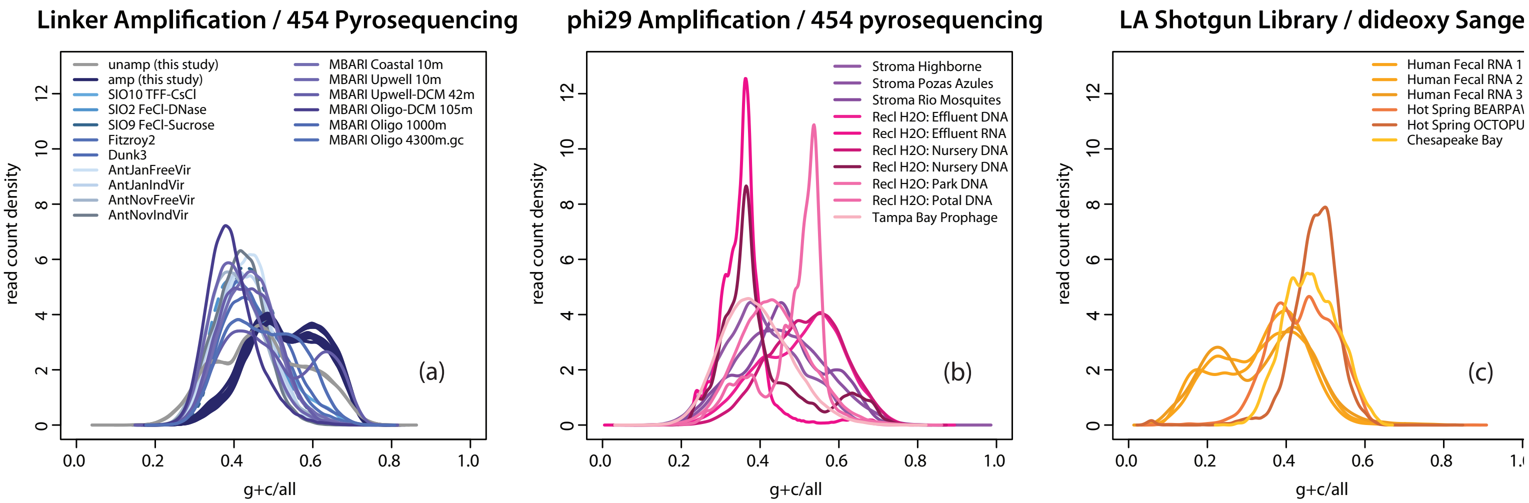

Supplement: Supplementary file 2 [file emi0014-2526-SD2.pdf]
