# Supplementary material for: Towards quantitative metagenomics of wild viruses and other ultra-low concentration DNA samples: a rigorous assessment and optimization of the linker amplification method
Source: Environ Microbiol. 2012 Sep;14(9):2526–37. doi: 10.1111/j.1462-2920.2012.02791.x (PMC3466414; doi:10.1111/j.1462-2920.2012.02791.x)

Supplementary Figure 3. The magnitude of difference in %G+C bias between unamplified and amplified treatments was assessed by the magnitude of difference between the integrated area under their %G+C-bias plot curves (Figure 3). As cycle number increased (and quantity of starting material decreased), there was a slight trend in the deviation of amplified from unamplified treatments.

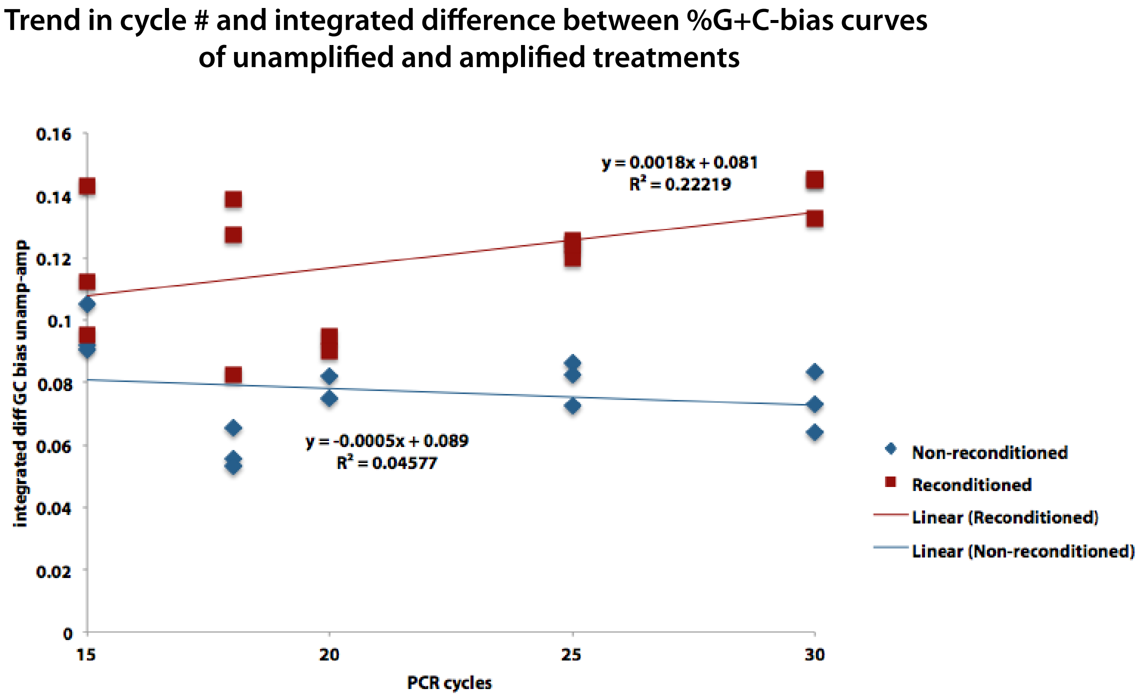

Supplement: Supplementary file 3 [file emi0014-2526-SD3.pdf]
