# Supplementary material for: Towards quantitative metagenomics of wild viruses and other ultra-low concentration DNA samples: a rigorous assessment and optimization of the linker amplification method
Source: Environ Microbiol. 2012 Sep;14(9):2526–37. doi: 10.1111/j.1462-2920.2012.02791.x (PMC3466414; doi:10.1111/j.1462-2920.2012.02791.x)

Supplementary Figure 4. Quality score profile of Illumina reads from 20 pooled freshwater cyanophage genomes (Deng and Sullivan, unpublished) generated from a linker amplified library. Boxplot generated by Fastx Toolbox.

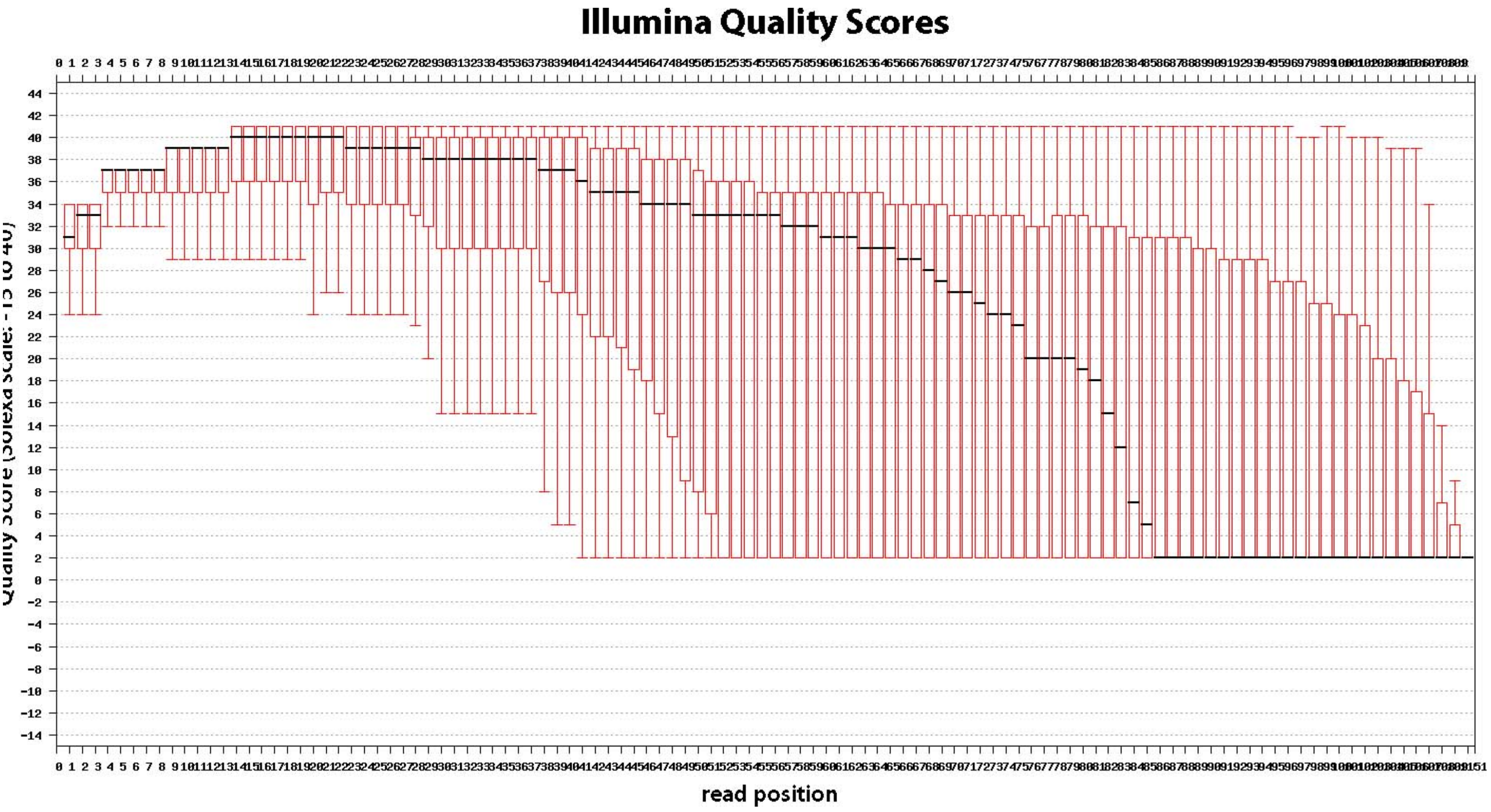

Supplement: Supplementary file 4 [file emi0014-2526-SD4.pdf]
