# Supplementary material for: Towards quantitative metagenomics of wild viruses and other ultra-low concentration DNA samples: a rigorous assessment and optimization of the linker amplification method
Source: Environ Microbiol. 2012 Sep;14(9):2526–37. doi: 10.1111/j.1462-2920.2012.02791.x (PMC3466414; doi:10.1111/j.1462-2920.2012.02791.x)

Supplementary Figure 5. Log-linear relationship between PCR cycle numbers and starting DNA, as it is diluted to extinction. Test was performed with dilution of a single phage genome, H105/1.

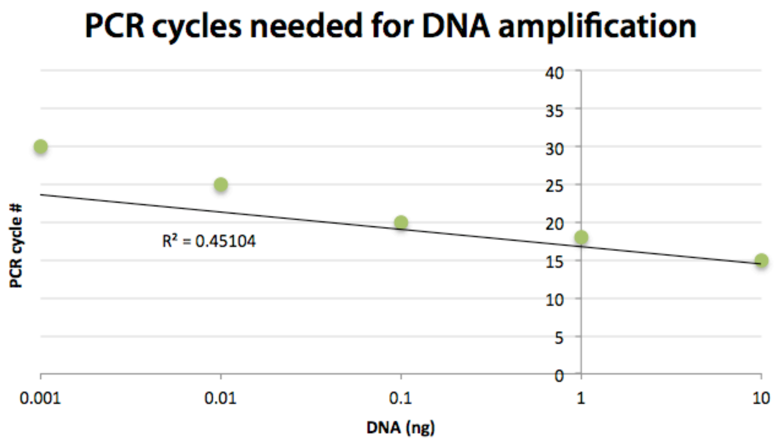

Supplement: Supplementary file 5 [file emi0014-2526-SD5.pdf]
