# Supplementary material for: Towards quantitative metagenomics of wild viruses and other ultra-low concentration DNA samples: a rigorous assessment and optimization of the linker amplification method
Source: Environ Microbiol. 2012 Sep;14(9):2526–37. doi: 10.1111/j.1462-2920.2012.02791.x (PMC3466414; doi:10.1111/j.1462-2920.2012.02791.x)
Supplement: Supplementary file 6 [file emi0014-2526-SD6.pdf]

Supplementary Table 1. Detailed break-down of linker amplification method, including time and cost estimates from sample-to-sequence for a typical 20 L aquatic virus sample.

**1.**	**Ligation**	*time/sample*	*$/sample*	*kit used*
a	Ultrasonic shearing of DNA to 400-800 bp, Covaris	1 week*	$20**	*time depends on turn-around of facility, actually process approx. 5 min; **price includes Agilent Bioanalyzer fragment sizing
b	Concentrate Sheared DNA	15-20 min	$3.40	Amicon Ultra-0.5ml 30K (UFC503096)
c	End repair DNA	1 hr	$5	Epicentre Biotechnologies
d	Clean-up reaction	10 min	$2.30	Qiagen MinElute or QiaQuick (#28104)
e	Ligate Fwd and Rev linker to DNA	2 hr	$2.40	Fast-Link Ligation Kit, Epicentre (LK0750)
f	Clean-up reaction	10 min	$2.30	Qiagen MinElute or QiaQuick
f-i	Size Fractionation: SPRI Beads	20 min	$0.85	Agencourt AMPure XP; Beckman Coulter A63880
	or			
f-ii	Size Fractionation: gel-sizing	3 hr	$1.40	Seakem GTG Agarose
	DNA recovery: gel extraction	0.5 hr	$2.20	QiaQuick MinElute gel extraction kit
	or			
f-iii	Size Fractionation: Pippin Prep	5 min	$11.25	Sage Science cassette kit
**2.**	**Amplification**	*time/sample*	*$/sample*	*kit used*
a	Small-scale PCR titration: determine optimal cycle #	1-2hr	$26.30	LA TaKaRa HS polymerase
b	Large-scale PCR	1-2hr		
c	Reconditioning PCR	0.5 hr		
d	PCR clean-up	10 min	$2.50	Qiagen MinElute or QiaQuick
e	Pico Green Quantification	0.5hr	$1.00	
**TOTAL**		**1-2 days work (1 week wait*)**	**$67.50**	
