# Supplementary material for: Towards quantitative metagenomics of wild viruses and other ultra-low concentration DNA samples: a rigorous assessment and optimization of the linker amplification method
Source: Environ Microbiol. 2012 Sep;14(9):2526–37. doi: 10.1111/j.1462-2920.2012.02791.x (PMC3466414; doi:10.1111/j.1462-2920.2012.02791.x)
Supplement: Supplementary file 7 [file emi0014-2526-SD7.pdf]

Supplementary Table 2. Comparison of DNA polymerase sensitivity and specificity. PFU Turbo HotStart out-performed LA-TaKaRa in 13 virus samples (blue), while the opposite held for one microbial sample (orange). However, the sensitivity comes at a cost, as PFU 'no template control' amplified at 30 and 35 cycles (red), while TaKaRa did not. All samples had been subjected to the LA protocol through ligation to Linker A and amplification (Supp. Table 1). +, amplification confirmed by strong band in post-PCR gel; −, no detectable amplification; ±, faint band indicating small degree of amplification; n.d., no data. ++microbial samples were sheared by nebulization, rather than Covaris; **below detection limit of PicoGreen assay, which in practice is 1-5 pg.

	**Sample**	**Starting DNA (pg/µl)**	*20 cycles*		*25 cycles*		*30 cycles*		*35 cycles*	
			**PFU**	**TAK**	**PFU**	**TAK**	**PFU**	**TAK**	**PFU**	**TAK**
Trial 1	Tara #23 DCM 1:100	229	+	+	+	+	+	+	+	+
	Tara #30 DCM 1:100	1495	+	+	+	+	+	+	+	+
	Fitzroy viral 1:100	1.9	+	+	+	+	+	+	+	+
	Dunk viral 1:100	4.9	+	+	+	+	+	+	+	+
	Sept/09 B2O micro (16S) 1:100++	17.4	+	+	+	+	+	+	+	+
	Aug/09 P26 10m	< 1 **	+	+	+	+	+	+	+	+
	Jun/09 P26 10m	< 1	+	+	+	+	+	+	+	+
	Jun/09 P4 10m 1:10	855	+	+	+	+	+	+	+	+
	Jun/08 P26 10m	6.3	−	−	±	±	+	+	+	+
	Feb/09 P26 1000m	6	−	−	+	±	+	+	+	+
	Jun/09 P4 500m	2.2	−	−	+	±	+	+	+	+
	Jun/09 P12 2000m	< 1	−	−	−	−	+	−	+	+
	Dec/09 Line67 Open DCM	872	+	+	+	+	+	+	+	+
	Apr/09 SIO Rep 3-NT	175	±	±	+	+	+	+	+	+
	No Template Control	n.a.	−	−	−	−	+	−	+	−
Trial 2	TUSD #20 1:30	< 1	−	−	+	−	+	+	+	+
	TUSD #20 1:300	< 0.1	−	−	−	−	+	±	+	+
	TUSD #23 1:30	80.4	−	−	±	−	+	+	+	+

	TUSD #23 1:300	8.4	–	–	–	–	+	–	+	+
	Jun/08 P26-10m	< 1	–	–	+	–	+	+	+	+
	Feb/09 P26 2000m	3.7	–	–	+	–	+	+	+	+
	Aug/08 P26 10m	< 1	–	–	+	–	+	+	+	+
	Aug/08 P26 1000m	< 1	–	–	–	–	+	±	+	+
	Feb/09 P26 10m	3.2	–	–	+	±	+	+	+	+
	Jun/08 P26 2000m	< 1	–	–	–	–	+	–	+	+
	No Template Control	n.a.	–	–	–	–	–	–	–	–
	Sept/09 B2 micro (16S)	28	–	±	n.d.	+	–	+	–	+
	Jun/10 SIO micro 1:100 (16S)	638	–	–	±	±	+	+	+	+
Trial 3	Jun/10 SIO micro 1:1000 (16S)	1.9	–	–	–	–	±	±	+	+
	Dec/09 Fitzroy micro 1:10	2900	+	+	+	+	+	+	+	+
	Dec/09 Dunk micro 1:200	1220	+	+	+	+	+	+	+	+
	No Template Control	n.a.	n.d.	n.d.	–	–	n.d.	n.d.	–	–
	TUSD phage mix (n = 2)	100000	n.d.	n.d.	+	+	+	+	+	+
	TUSD phage mix (n = 2)	10000	n.d.	n.d.	+	+	+	+	+	+
Trial 4	TUSD phage mix (n = 2)	3300	n.d.	n.d.	±	+	+	+	+	+
	TUSD phage mix (n = 2)	1000	n.d.	n.d.	–	±	+	+	+	+
	TUSD phage mix (n = 2)	200	n.d.	n.d.	–	–	±	+	+	+
	No Template Control	n.a.	n.d.	n.d.	–	–	–	–	–	–
