# Supplementary material for: Towards quantitative metagenomics of wild viruses and other ultra-low concentration DNA samples: a rigorous assessment and optimization of the linker amplification method
Source: Environ Microbiol. 2012 Sep;14(9):2526–37. doi: 10.1111/j.1462-2920.2012.02791.x (PMC3466414; doi:10.1111/j.1462-2920.2012.02791.x)
Supplement: Supplementary file 8 [file emi0014-2526-SD8.pdf]

Supplementary Table 3. Comparison of sheared DNA size-fractionation techniques. All data are post-size selection and determined by the Agilent Bioanalyzer. The three tests were performed with 13.7 µl of DNA (183 ng DNA total) from the same pool of sheared starting DNA (Fig. 2a)

**Sample**	**Volume recovered in target 400-600 bp range (µl)**	**[DNA] recovered in target 400-600 bp range (ng/µl)**	**DNA recovered in target 400-600 bp range (ng)**	**% recovery of sheared starting DNA (183 ng start)**	***TARGET RECOVERY EFFICIENCY*** **(% DNA recovered in 400-600 bp)**	**Actual size range recovered (*Fig. 2b*)**
Pippin Prep A	42	1.27	53.34	29	94	
Pippin Prep B	39	1.24	48.36	26	95	*400-600 bp*
Pippin Prep C	39	1.37	53.43	29	96	
Standard Gel 1A	20	3.95	79	43	64	
Standard Gel 2A	20	4.4	88	48	74	*400-750 bp*
Standard Gel 3A	20	4.39	87.8	48	72	
Ampure Bead 1	20	5.91	118.2	65	49	
Ampure Bead 2	20	5.05	101	55	46	*400-950 bp*
Ampure Bead 3	20	5.34	106.8	58	50	
