# Supplementary material for: Towards quantitative metagenomics of wild viruses and other ultra-low concentration DNA samples: a rigorous assessment and optimization of the linker amplification method
Source: Environ Microbiol. 2012 Sep;14(9):2526–37. doi: 10.1111/j.1462-2920.2012.02791.x (PMC3466414; doi:10.1111/j.1462-2920.2012.02791.x)
Supplement: Supplementary file 9 [file emi0014-2526-SD9.pdf]

Supplementary Table 4. Evaluation of Linker Amplification efficiency. Percent DNA recovery (post-shearing) of the concentration, blunt-end repair/reaction clean-up, Linker-A ligation/reaction clean-up, and size fractionation (gel)/recovery of DNA from gel. This is the template DNA used in PCR amplification.

Group	Sample ID	ng DNA sheared	ng DNA recovered	% recovery
Environmental samples	SIO-3 NT	151.7	13.42	8.8
	SIO-4 NT	148.5	15.78	10.6
	SIO-3 CsCl	150.4	9.8	6.5
	SIO-4 CsCl	129.6	8.09	6.2
	SIO-3 sucrose	148.8	7.7	5.2
	SIO-4 sucrose	149.9		3.5
	TUSD #20a	149.3	5.32	3.6
Clonal phage lysates	TUSD #20b	49.8	2.11	4.2
	TUSD #21	151.2	7.64	5.1
	TUSD #22	149	6.27	4.2
	TUSD #23a	148.5	6.3	4.2
	TUSD #23b	54	1.86	3.4
	TUSD #24	149.6	8.22	5.5
	PSS-2	42.4	3.87	9.1
	**Avg. ± s.d.**	54	1.86	**5.7 ± 2.3**
