# Supplementary material for: Towards quantitative metagenomics of wild viruses and other ultra-low concentration DNA samples: a rigorous assessment and optimization of the linker amplification method
Source: Environ Microbiol. 2012 Sep;14(9):2526–37. doi: 10.1111/j.1462-2920.2012.02791.x (PMC3466414; doi:10.1111/j.1462-2920.2012.02791.x)
Supplement: Supplementary file 10 [file emi0014-2526-SD10.pdf]

Supplementary Table 5. Increase in DNA as a result of Linker Amplification, from both environmental and phage lysate DNA preps. Starting DNA concentration reflects that after the shearing and linker ligation steps.

	Sample	DNA ng/ul	ng DNA (in 20 µl PCR)[1]	ng DNA after PCR[2]	fold-increase
Environmental	SIO-3 NT	0.34	6.8	88.53	13
	SIO-4 NT	0.39	7.8	165.43	21
	SIO-3 CsCl	0.25	5	51.74	10
	SIO-4 CsCl	0.21	4.2	46.15	11
	SIO-3 sucrose	0.19	3.8	1146.53	302
	SIO-4 sucrose	0.25	5	123.93	25
Clonal lysate	TUSD #20	0.05	1	1197.17	1197
	TUSD #21	0.22	4.4	1161.31	264
	TUSD #22	0.18	3.6	1361.94	378
	TUSD #23	0.1	2	1287.34	644
	TUSD #24	0.23	4.6	1218.65	265
	Phage PSS2	0.05	1	1235.82	1236

[1] PCR reactions run with **PFU-Turbo Hot Start** for 25 cycles

[2] PCR product pooled, purified.
