# Supplementary material for: Towards quantitative metagenomics of wild viruses and other ultra-low concentration DNA samples: a rigorous assessment and optimization of the linker amplification method
Source: Environ Microbiol. 2012 Sep;14(9):2526–37. doi: 10.1111/j.1462-2920.2012.02791.x (PMC3466414; doi:10.1111/j.1462-2920.2012.02791.x)
Supplement: Supplementary file 11 [file emi0014-2526-SD11.pdf]

Supplementary Table 6. Two-tailed paired Student's t-tests comparing the integrated areas between unamplified read depth curve and curve of each amplified treatment. Probabilities are reported. To test for the effect of cycle number on degree of deviation from unamplified read depth, a test was performed for each combination of cycle numbers; none were significant, with all $p > 0.05$.

All treatments

cycles	*15*	*18*	*20*	*25*	*30*
15					
18	*0.819*				
20	*n.a.*	*n.a.*			
25	*0.212*	*0.233*	*n.a.*		
30	*0.103*	*0.337*	*n.a.*	*0.611*	

Non-reconditioned

cycles	*15*	*18*	*20*	*25*	*30*
15					
18	*0.225*				
20	*n.a.*	*n.a.*			
25	*0.239*	*0.188*	*n.a.*		
30	*0.168*	*0.304*	*n.a.*	*0.446*	

Reconditioned

cycles	*15*	*18*	*20*	*25*	*30*
15					
18	*0.282*				
20	*n.a.*	*n.a.*			
25	*0.718*	*0.131*	*n.a.*		
30	*0.556*	*0.136*	*n.a.*	*0.926*	
