# Supplementary material for: Towards quantitative metagenomics of wild viruses and other ultra-low concentration DNA samples: a rigorous assessment and optimization of the linker amplification method
Source: Environ Microbiol. 2012 Sep;14(9):2526–37. doi: 10.1111/j.1462-2920.2012.02791.x (PMC3466414; doi:10.1111/j.1462-2920.2012.02791.x)
Supplement: Supplementary file 12 [file emi0014-2526-SD12.pdf]

Supplementary Table 7. Two-tailed paired Student's t-tests comparing the integrated areas between unamplified %G+C bias curve of unamplified and each amplified treatment for H105/1. Significant (p <0.05) values are yellow; highly significant (p <0.001) values are orange. "n.a." indicates not enough data to test, as the 20-cycle treatment was dropped from the analysis due to failed sequencing.
(a) Probability that differences between cycle numbers and starting DNA amounts is due to chance.
(b) Probability that differences between reconditioned and non-reconditioned samples is due to chance. All reconditioned samples are compared to all non-reconditioned ("all treatments"); reconditioning is also tested within replicates of each cycle number.

(a) **All data: %G+C range 0.31-0.55**

All treatments (n = 6 for all)

cycles	*15*	*18*	*20*	*25*	*30*
15					
18	*0.279*				
20	*n.a.*	*n.a.*			
25	*0.641*	*0.151*	*n.a.*		
30	*0.954*	*0.037*	*n.a.*	*0.444*	

Non-reconditioned (n = 3 for all)

cycles	*15*	*18*	*20*	*25*	*30*
15					
18	*0.001*				
20	*n.a.*	*n.a.*			
25	*0.211*	*0.102*	*n.a.*	*n.a.*	
30	*0.101*	*0.175*	*n.a.*	*0.460*	

Reconditioned (n = 3 for all)

cycles	*15*	*18*	*20*	*25*	*30*
15					
18	*0.986*				
20	*n.a.*	*n.a.*			
25	*0.726*	*0.706*	*n.a.*		
30	*0.312*	*0.204*	*n.a.*	*0.021*	

(b) **All data: %G+C range 0.31-0.55**

Non-recond. vs. Reconditioned	
all treatments (n = 14)	4.2E-05
15 cycles, 10 ng (n = 3)	*0.314*
18 cycles, 1 ng (n = 3)	*0.057*
20 cycles, 0.1 ng (n = 3)	*n.a.*
25 cycles, 10 pg (n = 3)	*0.010*
30 cycles, 1 ng (n = 3)	*0.019*
